# Two genetic loci associated with ankle injury

**DOI:** 10.1371/journal.pone.0185355

**Published:** 2017-09-28

**Authors:** Stuart K. Kim, John P. Kleimeyer, Marwa A. Ahmed, Andrew L. Avins, Michael Fredericson, Jason L. Dragoo, John P. A. Ioannidis

**Affiliations:** 1 Dept. Developmental Biology, Stanford University Medical Center, Stanford, CA, United States of America; 2 Dept. Orthopaedic Surgery, Stanford University Medical Center, Stanford, CA, United States of America; 3 Dept. Physical Medicine and Rehabilitation, Harvard Medical School, Boston, MA, United States of America; 4 Kaiser Permanente Northern California, Division of Research, Oakland, CA, United States of America; 5 Dept. of Medicine, Stanford Prevention Research Center, Stanford University School of Medicine, Stanford, CA, United States of America; 6 Dept. of Health Research and Policy, Division of Epidemiology, Stanford University School of Medicine, Stanford, CA, United States of America; 7 Dept. of Statistics, Stanford University School of Humanities and Sciences, Stanford, CA, United States of America; Kunming Institute of Zoology, Chinese Academy of Sciences, CHINA

## Abstract

Ankle injuries, including sprains, strains and other joint derangements and instability, are common, especially for athletes involved in indoor court or jumping sports. Identifying genetic loci associated with these ankle injuries could shed light on their etiologies. A genome-wide association screen was performed using publicly available data from the Research Program in Genes, Environment and Health (RPGEH) including 1,694 cases of ankle injury and 97,646 controls. An indel (chr21:47156779:D) that lies close to a collagen gene, *COL18A1*, showed an association with ankle injury at genome-wide significance (p = 3.8x10^-8^; OR = 1.99; 95% CI = 1.75–2.23). A second DNA variant (rs13286037 on chromosome 9) that lies within an intron of the transcription factor gene *NFIB* showed an association that was nearly genome-wide significant (p = 5.1x10^-8^; OR = 1.63; 95% CI = 1.46–1.80). The *ACTN3* R577X mutation was previously reported to show an association with acute ankle sprains, but did not show an association in this cohort. This study is the first genome-wide screen for ankle injury that yields insights regarding the genetic etiology of ankle injuries and provides DNA markers with the potential to inform athletes about their genetic risk for ankle injury.

## Introduction

Ankle sprains and strains are the most common musculoskeletal injuries in athletes, especially indoor or court sports [1–3]. Low ankle sprains occur with injury to ankle ligaments, most commonly the lateral ligament complex. Ankle strains describe pathologic stretching or tearing of muscle or tendon. Other ankle joint derangements, such as instability, may occur with insufficiency of the soft tissue restraints of the ankle or malalignment. These injuries (collectively referred to as ankle injuries) often occur in athletic activity with axial loading of an inverted, plantar-flexed foot as the most common mechanism. Ankle sprains are more common in women than men, and in children or adolescents compared to adults [2,3].

Little is known about genetic factors that affect risk for ankle injury. One study reported an association of the R577X mutation in the *actinin 3* (*ACTN3*) gene with acute ankle sprain from a study involving 142 cases of injury (p = .011)[4]. *ACTN3* encodes alpha-actinin skeletal muscle isoform 3, which is an actin-binding protein expressed in all skeletal muscles. In principle, genetic studies such as this have the potential to identify contributing factors to ankle injuries and to provide diagnostic markers informing individuals about their personal risk for injury.

In order to identify genetic factors that may provide insight about ankle injuries, we screened the entire genome for loci associated with these injuries. We identified individuals who had suffered an ankle injury from a cohort of 99,342 patients in the Research Program on Genes, Environment, and Health (RPGEH) of the Kaiser Permanente, Northern California (KPNC) health plan. A gene association analysis revealed one locus on chromosome 21 associated with ankle injury with genome-wide significance, and a second locus on chromosome 9 with an association just below genome-wide significance. We re-tested the *ACTN3* SNP for association with ankle injury in our cohort but did not see a significant association.

## Methods

A genome-wide association screen was performed for ankle injury using data from the genotyped Genetic Epidemiology Research on Adult Health and Aging (GERA) cohort of the Research Program in Genes, Environment and Health (RPGEH). The data generation and data analysis pipeline have been previously described [5]. A complete description of the cohort and study design can be found in dbGaP (Study Accession: phs000674.v1.p1).

Our analysis cohort (n = 99,342) includes 57,606 females, 41,670 males, and 66 individuals of uncertain sex (Table 1). Sex was determined previously based on heterozygosity of the X chromosome (dbGAP Study Accession: phs000674.v1.p1). Moreover, our analysis cohort is ethnically diverse, including 83,264 European-White (EUR); 8,560 Latino (LAT) and 7,518 East Asian (EAS) individuals based on ancestry principle components.

**Table 1:** Demographic factors of the GERA study population used in genome-wide association analyses of ankle injury.

	Cases^a^	Controls	Overall
**Subjects** (%)	1,696 (1.7%)	97,646 (98.3%)	99,342
**Sex** (%)^**b**^			
Female	1,049 (1.8%)	56,557 (98.2%)	57,606
Male	645 (1.5%)	41,025 (98.5%)	41,670
Undetermined	1 (1.5%)	65 (98.5%)	66
**Ancestry** (%)^**c**^			
European	1,421 (1.7%)	81,843 (98.3%)	83,264
Latin American	154 (1.8%)	8,406 (98.2%)	8,560
East Asian	121 (1.6%)	7,397 (98.4%)	7,518
**Age**^**d**^	60.1 (±13.1)	62.9 (±13.7)	62.8 (±13.7)

^a^ Cases with ankle injury as defined by individuals with one or more qualifying ICD-9, ICD-10 or CPT-4 code in their EHR. For details, see Methods.

^b^ Sex/gender as determined by an individual’s genetic data, reported as the number and percentage of total.

^c^ Race/ethnic groups as determined by PCA on an individual’s genetic data from the GERA cohort. Reported as the number and percentage of total for each respective group.

^d^ Age at subject enrollment in the GERA cohort, reported as mean age with standard deviation.

Participants were genotyped at over 650,000 SNPs [6]. Genotypes were then imputed using standard procedures with a cutoff of R^2^ > 0.3 [7–9]. The quality of the imputed data was previously validated in Jorgenson et al., 2015 [10].

Determination of genetic ancestry was performed by principal component analysis (PCA), as previously described [5,11]. These ancestry principal components were used in the GWAS to adjust for genetic ancestry.

### Phenotype definition

Ankle injury cases were identified in the GERA cohort based on clinical diagnoses and surgical procedures captured in the KPNC electronic health record system. The electronic health record includes reported injuries over the entire lifetime of the patients, including those that occurred prior to enrollment in KPNC as well as those that occurred after the genotyping analysis was performed, if reported by the patient and recorded by the physician. International Classification of Disease, Ninth Revision (ICD-9), International Classification of Disease, Tenth Revision (ICD-10) and Common Procedure Terminology, Fourth Edition (CPT-4) codes, were used to identify cases of ankle injury (Table 2). Table 2 includes codes for: 1) ankle sprain, 2) ankle strain, 3) surgical repair for disrupted ankle ligament and 4) joint derangement of the ankle or foot. Ankle sprains and strains are not differentiated in the ICD-9 codes, while they are in the ICD-10 codes. Joint derangement includes joint instability.

**Table 2:** Ankle injury phenotypes classified by ICD and/or CPT codes.

ICD-9 code	Code Description^a^	N
845.01	Sprain/Strain Deltoid Ligament	38
845.02	Sprain/Strain Calcaneofibular Ligament	21
845.03	Sprain/Strain Tibiofibular Ligament, Distal	34
845.09	Other Sprains/Strains Of Ankle	789
718.87	Other Joint Derangement, Not Elsewhere Classified, Ankle And Foot	444
ICD-10 code		
S93.401A	Sprain Of Unspecified Ligament Of Right Ankle, Initial Encounter	190
S93.401D	Sprain Of Unspecified Ligament Of Right Ankle, Subsequent Encounter	44
S93.401S	Sprain Of Unspecified Ligament Of Right Ankle, Sequela	1
S93.402A	Sprain Of Unspecified Ligament Of Left Ankle, Initial Encounter	165
S93.402D	Sprain Of Unspecified Ligament Of Left Ankle, Subsequent Encounter	45
S93.402S	Sprain Of Unspecified Ligament Of Left Ankle, Sequela	1
S93.409A	Sprain Of Unspecified Ligament Of Unspecified Ankle, Initial Encounter	3
S93.411A	Sprain Of Calcaneofibular Ligament Of Right Ankle, Initial Encounter	2
S93.412A	Sprain Of Calcaneofibular Ligament Of Left Ankle, Initial Encounter	2
S93.421D	Sprain Of Deltoid Ligament Of Right Ankle, Subsequent Encounter	1
S93.422A	Sprain Of Deltoid Ligament Of Left Ankle, Initial Encounter	1
S93.422D	Sprain Of Deltoid Ligament Of Left Ankle, Subsequent Encounter	2
S93.422S	Sprain Of Deltoid Ligament Of Left Ankle, Sequela	1
S93.431A	Sprain Of Tibiofibular Ligament Of Right Ankle, Initial Encounter	1
S93.432D	Sprain Of Tibiofibular Ligament Of Left Ankle, Subsequent Encounter	1
S93.432S	Sprain Of Tibiofibular Ligament Of Left Ankle, Sequela	1
S93.492A	Sprain Of Other Ligament Of Left Ankle, Initial Encounter	4
S93.492D	Sprain Of Other Ligament Of Left Ankle, Subsequent Encounter	1
S93.492S	Sprain Of Other Ligament Of Left Ankle, Sequela	1
CPT		
27695	Repair, Primary, Disrupted Ligament, Ankle; Collateral	15

^a^ International Statistical Classification of Diseases and Related Health Problems (ICD-9 or ICD-10) and Current Procedural Terminology (CPT-4) codes extracted from KPNC electronic health records of GERA cohort subjects.

### Genome-wide association and meta-analysis

Genome-wide association analyses of the GERA cohort genomic data were conducted as previously described [5,11]. SNP associations were tested with ankle injury with a logistic regression model using allele counts for typed and imputed SNPs in an additive genetic model for each of the race/ethnic populations. The model was adjusted for genetic sex, age at enrollment into the RPGEH cohort, race/ethnicity using principal components, and variations in genotyping protocol [5,11]. We used 10 principal components for European (EUR), 6 for Latin American (LAT) and 3 for East Asian (EAS). The final number of SNPs that were analyzed was 8,795,348 for EUR; 9,153,118 for LAT and 8,055,053 for EAS. To account for inflation due to population stratification, the genomic control parameter (λ_gc_) was calculated: EUR (1.008), LAT (1.008), EAS (1.048). Subsequently, p-values were adjusted for genomic control in each population. Results from each population were combined by inverse-variance, fixed-effects meta-analysis as previously described [5,11]. SNPs that did not contain data for EUR were removed, as EUR comprises more than 80% of the cohort. The final number of SNPs that was analyzed in the fixed-effects meta-analysis was 8,183,964. Power calculations were made using the software at http://csg.sph.umich.edu/abecasis/cats/gas_power_calculator/index.html [12].

We examined the level of heterogeneity as previously described [5,11].

To perform the sensitivity analysis, the total set of 1696 cases was split into a subset that are known to involve an ankle injury (1275 cases) and another subset (421 cases) that are either ankle or foot derangements as they were identified solely by ICD-9 code 718.87 (Other Joint Derangement, Not Elsewhere Classified, Ankle And Foot). Logistic regression was used to calculate the association of chr21:47156779:D and rs13286037 with each subset of cases. Because the number of cases in the subgroups was smaller than in the total, only six principal components were used for the EUR ancestry group. P-values were calculated from a fixed-effects meta-analysis.

Further bioinformatics investigation of the top genome-wide significant loci from the meta-analysis was conducted as previously described [5,11].

Summary statistics for all SNPs from the fixed-effects meta-analysis are available at NIH GRASP: https://grasp.nhlbi.nih.gov/FullResults.aspx.

### Ethical considerations

This study analyzed stored data from RPGEH subjects who consented to genomic testing and use of their genomic data, as well as health data from the KPNC electronic health record, for future research studies. The health and genotype data for the subjects were de-identified. All study procedures were approved by the Institutional Review Board of the Kaiser Permanente Research Institute.

## Results

### Study population and genotype information

We performed a logistic regression for DNA variants associated with ankle injuries. Ankle injuries (which refers to ankle sprains, strains and other derangements) were identified by ICD-9, ICD-10 and CPT codes (Table 1). There were 1,696 cases and 97,646 controls in the GERA cohort. Overall, the period prevalence of ankle injury was 1.7%. Participation in sports was not included in the electronic health record, and hence we were not able to determine the incidence rate for the subset of the population who were athletes. Men showed a lower incidence of ankle injury than women that was statistically significant (p = 1.3x10^-3^; OR = .85; 95% CI = 0.77–0.94), consistent with previous results [2,3]. There was a small difference in the age of enrollment between the cases and controls (1.8 yrs) that was statistically significant (p = 1.5x10^-8^)(Table 1). One possibility is that this might be caused by an ascertainment bias where some elderly patients that enrolled in the RPGEH program might be systematically missed as cases if they incurred the ankle injury when they were young, before electronic records were in common practice.

### Genome-wide study for association with ankle injury

The RPGEH cohort, genotyping data, methodological approach and logical flow presented here overlap those used in previous work by the same authors on MCL injury, shoulder dislocation, plantar fasciitis, ACL injury and Achilles tendon injury [5,11,13,14]. However, the analyses presented here present new results and concepts on the genetic basis for ankle injury, which has not previously been analyzed at the genome-wide level.

**Fig 1:**
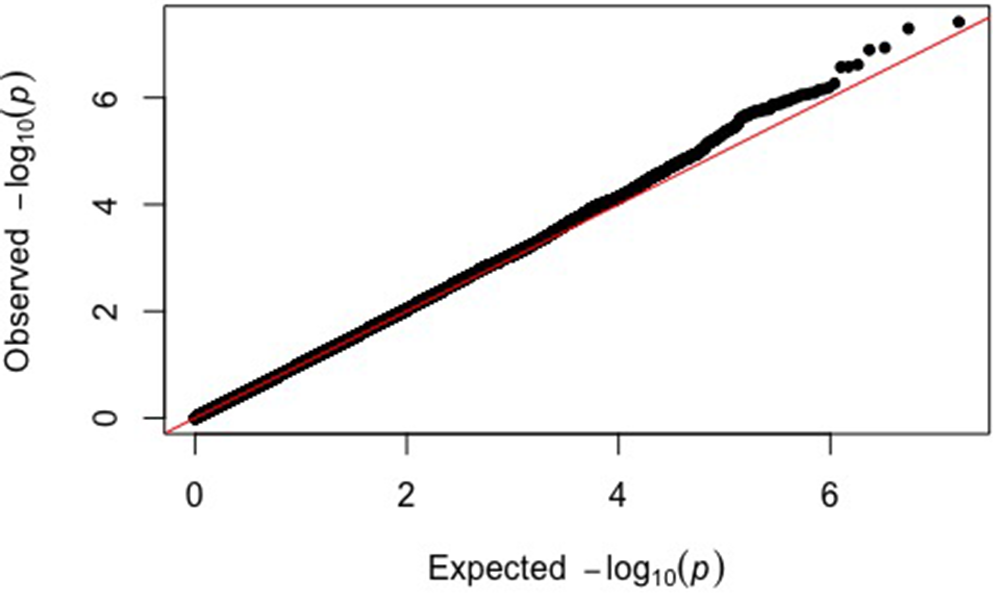
Quantile-quantile plot for genome-wide association analysis of ankle injury. The expected versus observed log transformed values for the 8,183,964 p-values from the meta-analysis are graphed. The y-axis shows the observed p-values and the x-axis shows the p-values expected by chance. The black dots represent the SNPs arranged by their observed p-values and the red line shows the expected trajectory if the SNPs had p-values expected by chance.

We compared the observed p-values to the distribution of p-values expected by chance in a Q-Q plot (Fig 1). The black dots deviate from the red line for the lowest observed p-values in the upper right hand corner, indicating that the observed association signals are significantly different than the signals that would be expected by chance.

**Fig 2:**
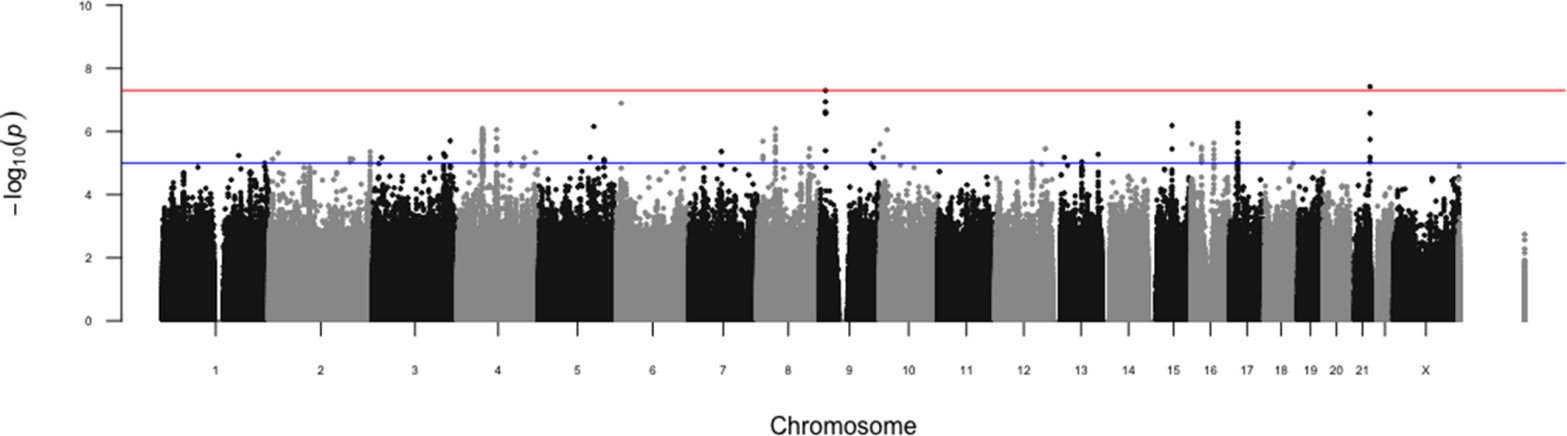
Manhattan plot for genome-wide association analysis of ankle injury. The -log_10_ p-values for association with ankle injury for SNPs from the meta-analysis are plotted by genomic position with chromosome number listed across the bottom. The y-axis shows the -log_10_ p-value for association with ankle injury. The blue line represents suggestive genome-wide significance (p<5x10^-5^) and the red line represents genome-wide significance (p<5x10^-8^).

The p-value for every SNP from the meta-analysis is shown in a Manhattan plot in Fig 2. Using p = 5x10^-8^ as a cut-off for genome-wide significance, chr21:47156779:D on chromosome 21 showed a genome-wide significant association with ankle injury (p = 3.8x10^-8^, Table 3). rs13286037 on chromosome 9 showed an association that was nearly genome-wide significant (5.1x10^-8^)(Table 3). For both chr21:47156779:D and rs13286037, the minor allele frequency was below 5%, meaning that there were a relatively small number of individuals carrying either one or two copies of the risk allele. Fisher’s exact test (i.e. linear discriminant analysis) is an alternative way to analyze the association of a DNA variant with ankle injury for small sample sizes. We repeated the analysis for association with ankle injury using Fisher’s exact test for these two DNA variants, and obtained p-values that were similar although slightly less strong than the values using logistic regression. Specifically, the p-values using Fisher’s exact test for chr21:47156779:D and rs13286037 were 4.8x10^-8^ and 9.5x10^-8^, respectively.

**Table 3:** Genome-wide association analyses for ankle injury.

Variant	Gene(s)	EA^a^	EAF^b^	P-value^c^	OR (95% CI)^d^
chr21:47156779:D^e^	*COL18A1*	A	.011	3.8x10^-8^	1.99 (1.75–2.23)
	*SLC19A1*				
	*PCBP3*				
rs13286037^e^	*NFIB*	A	.025	5.1x10^-8^	1.63 (1.46–1.80)

^a^ Effect allele (minor allele).

^b^ Effect allele frequency in the control population.

^c^ P-value from fixed-effects meta-analysis.

^d^ Adjusted allelic odds ratio with 95% confidence interval.

^e^ Genotypes for these SNPs were imputed: chr21:47156779:D (R^2^ = 0.73) and rs13286037 (R^2^ = 0.94).

Neither chr21:47156779:D nor rs13286037 were directly genotyped on the Affymetrix chips, but rather their genotype data was imputed (Table 3; S1 Table). For chr21:47156779:D, the R^2^ value was 0.73, indicating that the genotype was only partially accurate using imputation and thus care should be taken until true genotype data can be obtained. Inaccuracies in the genotype of chr21:47156779:D caused by imputation would not be expected to lead to a spurious association between chr21:47156779:D and ankle injury. Rather one might expect that the noise introduced by inaccurate imputation would weaken the association between the true genotype of chr21:47156779:D and ankle injury. For rs13286037, the R^2^ value was 0.94 indicating that the imputed genotype is fairly accurate.

Of the 1696 cases of ankle injury, 421 were identified based solely on ICD-9 code 718.87, which pertains to instability or hypermobility of joints in either the ankle or foot. For this code, most of the injuries involve the ankle but some may involve foot derangements instead. Because some of the diagnoses with this code may have involved a foot rather than an ankle derangement, we wanted to know whether the association of chr21:47156779:D and rs13286037 was similar between the subset of cases known to affect the ankle (1275 cases) versus the subset identified by ICD-9 718.87 (421 cases). We split the cases into the two sub-groups, then repeated the logistic regression and meta-analysis for each sub-group (Table 4). The results indicate that both chr21:47156779:D and rs13286037 show an association with both the ankle sprain/strain and the ankle/foot derangement phenotypes. The odds ratios are similar for the two phenotypes. As expected, the p-values become weaker as the number of cases drops in each sub-group. These results indicate that the association of the top two SNPs with the ankle sprain/strain and the ankle/foot derangement phenotypes are qualitatively similar.

**Table 4:** Sensitivity analysis for association with ankle sprains/strains and ankle/foot derangements.

chr21:47156779:D	Cases	P value ^a^	OR (95% CI) ^b^
All cases	1696	3.8x10^-8^	1.99 (1.75–2.23)
Ankle sprains/strains^c^	1275	1.2x10^-5^	1.89 (1.60–2.18)
Ankle/foot derangements^d^	421	5.2x10^-4^	2.26 (1.80–2.72)
rs13286037			
All cases	1696	5.1x10^-8^	1.63 (1.46–1.80)
Ankle sprains/strains^c^	1275	6.0x10^-7^	1.67 (1.47–1.87)
Ankle/foot derangements^d^	421	1.6x10^-2^	1.54 (1.19–1.89)

^a^ P-value from fixed-effects meta-analysis.

^b^ Adjusted allelic odds ratio with 95% confidence interval.

^c^Cases known to an ankle sprain or strain.

^d^Cases identified only by ICD-9 code 718.87 involving either an ankle or a foot derangement.

### Two loci associated with ankle injuries

For chr21:47156779:D, individuals that carried one copy of the risk allele (genotype A/AG) had a 1.86-fold increased risk of ankle injury compared to individuals with no risk alleles (genotype AG/AG)(Table 5). For rs13286037, individuals carrying one copy of the risk allele (A/T) had a 1.58-fold higher risk for ankle injury compared to individuals with no risk alleles (T/T)(Table 5). For both genetic variants, the risk was even higher in people carrying two copies of the risk allele, but there were too few such individuals for this result to be statistically significant (Table 5).

**Table 5:** Genotype distributions for chr21:47156779:D and rs13286037.

chr21:47156779:D	A/A	A/AG	AG/AG
Cases	1	67	1,585
Controls	8	2,091	92,988
Overall	9	2,158	94,573
Risk for ankle injury	0.111	.0310	.0167
Relative risk for ankle injury^a^ (95% CI)	6.65	1.86	1.00
	(0.84–52.36)	(1.44–2.37)	
rs13286037	A/A	A/T	T/T
Cases	3	131	1,612
Controls	83	4,845	95,394
Overall	86	4,976	97,006
Risk for ankle injury	.0361	.0263	.0166
Relative risk for ankle injury^a^ (95% CI)	2.17	1.58	1.00
	(0.69–6.89)	(1.35–1.90)	

^a^ Risk relative to individuals homozygous for the protective allele (95% CI).

The GWAS results were analyzed to determine whether the association with ankle injury for either chr21:47156779:D or rs13286037 was stronger in some ancestry groups than in others, a phenomenon known as heterogeneity [15]. Table 6 shows the p-values and odds ratios for these two SNPs for each ancestry group. The logistic regression analysis did not converge on a p-value for the EAS ancestry for either chr21:47156779:D or rs13286037 due to limited number of cases. As expected, the smallest p-value for both SNPs was observed for the European population, since 82% of the cohort was European. For the LAT ancestry group, the p-values were 0.94 for chr21:47156779:D and 0.08 for rs13286037. The odds ratios for each race were in the same direction and of similar magnitude. Using I^2^ and Cochran’s Q to assess heterogeneity, we saw no evidence of significant heterogeneity for rs13286037 between the EUR and LAT ancestry groups (Table 6). For chr21:47156779:D, the I^2^ estimate was 42%, suggesting that there might be heterogeneity between the EUR and LAT ancestry groups. However, the 95% confidence interval for I^2^ was 0–90, indicating that the presence and extent of heterogeneity is not certain.

**Table 6:** Association statistics for chr21:47156779:D and rs13286037 with ankle injury in individual ancestry groups.

Race	SNP	EA^a^	P-value^b^	OR	I^2^ ^d^	Q ^e^
				(95% CI)^c^		
EUR	chr21:47156779:D	A	1.57x10^-8^	2.07	42	0.19
				(1.61–2.66)	(0–90)	
LAT	chr21:47156779:D	A	0.94	1.04		
				(0.38–2.81)		
EAS	chr21:47156779:D	A	ND^f^	ND		
EUR	rs13286037	A	2.62x10^-7^	1.61	0	0.70
				(1.34–1.93)	(0–90)	
LAT	rs13286037	A	0.08	1.85		
				(.93–3.68)		
EAS	rs13286037	A	ND	ND		

^a^Effect allele.

^b^P value adjusted for lambda genomic inflation factor from fixed-effect meta-analysis.

^c^ Allelic odds ratio (95% confidence interval).

^d^ Percentage of variability between ancestry groups that is due to heterogeneity (95% confidence interval).

^e^ Cochran’s Q, p-value that the association is different between ancestry groups.

^f^No data.

**Fig 3:**
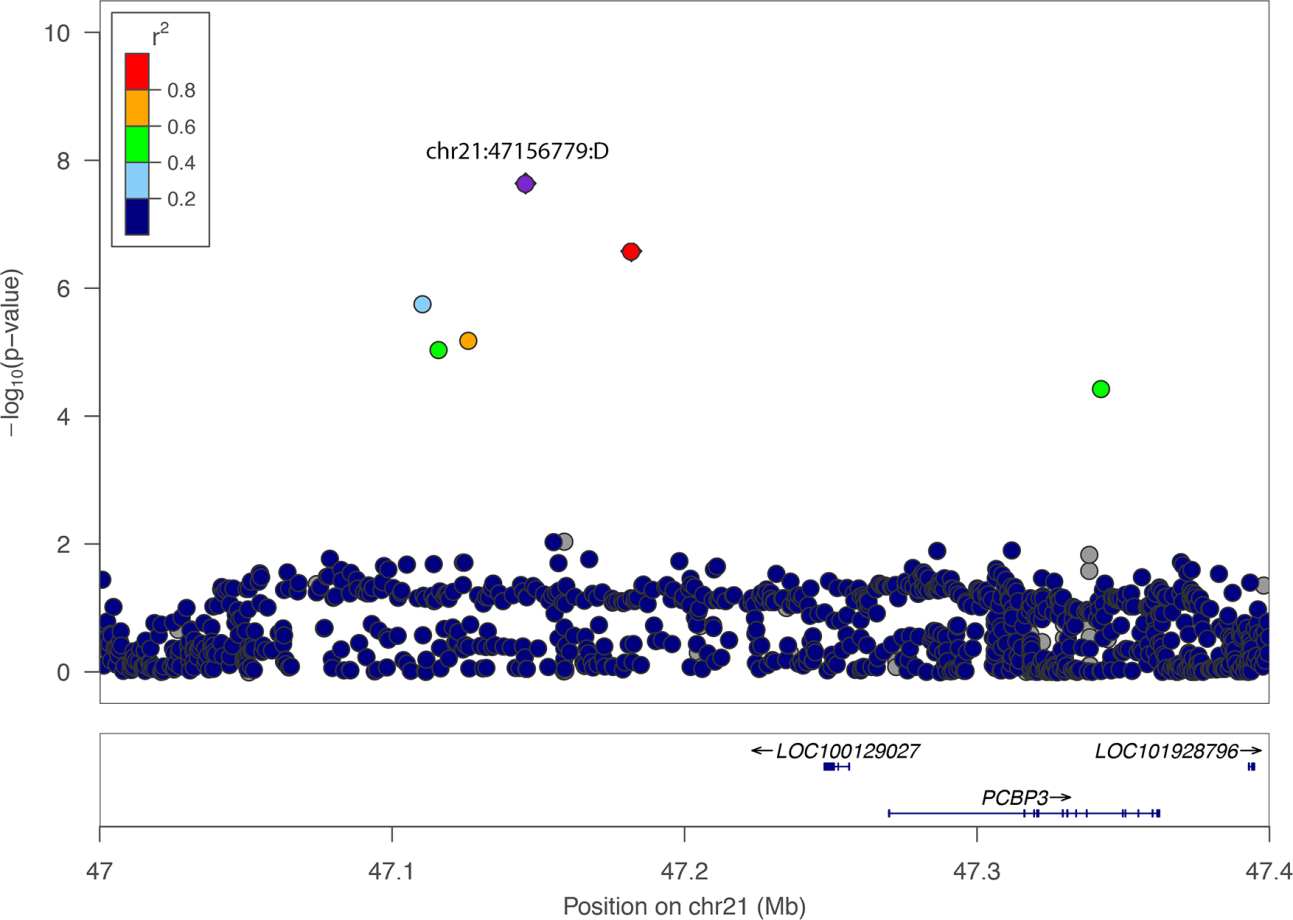
Regional-association plot for chr21:47156779:D with ankle injury. Tested SNPs are arranged by genomic position on chromosome 21 (x-axis) in a 600 kb window around the lead SNP chr21:47156779:D (purple diamond). The y-axis indicates -log_10_ p-values for association with ankle injury for each SNP. chr21:47156779:D is located in the intergenic region between *COL18A1/SLC19A1* and *PCBP3*. The location of *LINC00205* is not shown. The color of dots of the flanking SNPs indicates their linkage disequilibrium (R^2^) with the lead SNP as indicated by the heat map color key.

**Fig 4:**
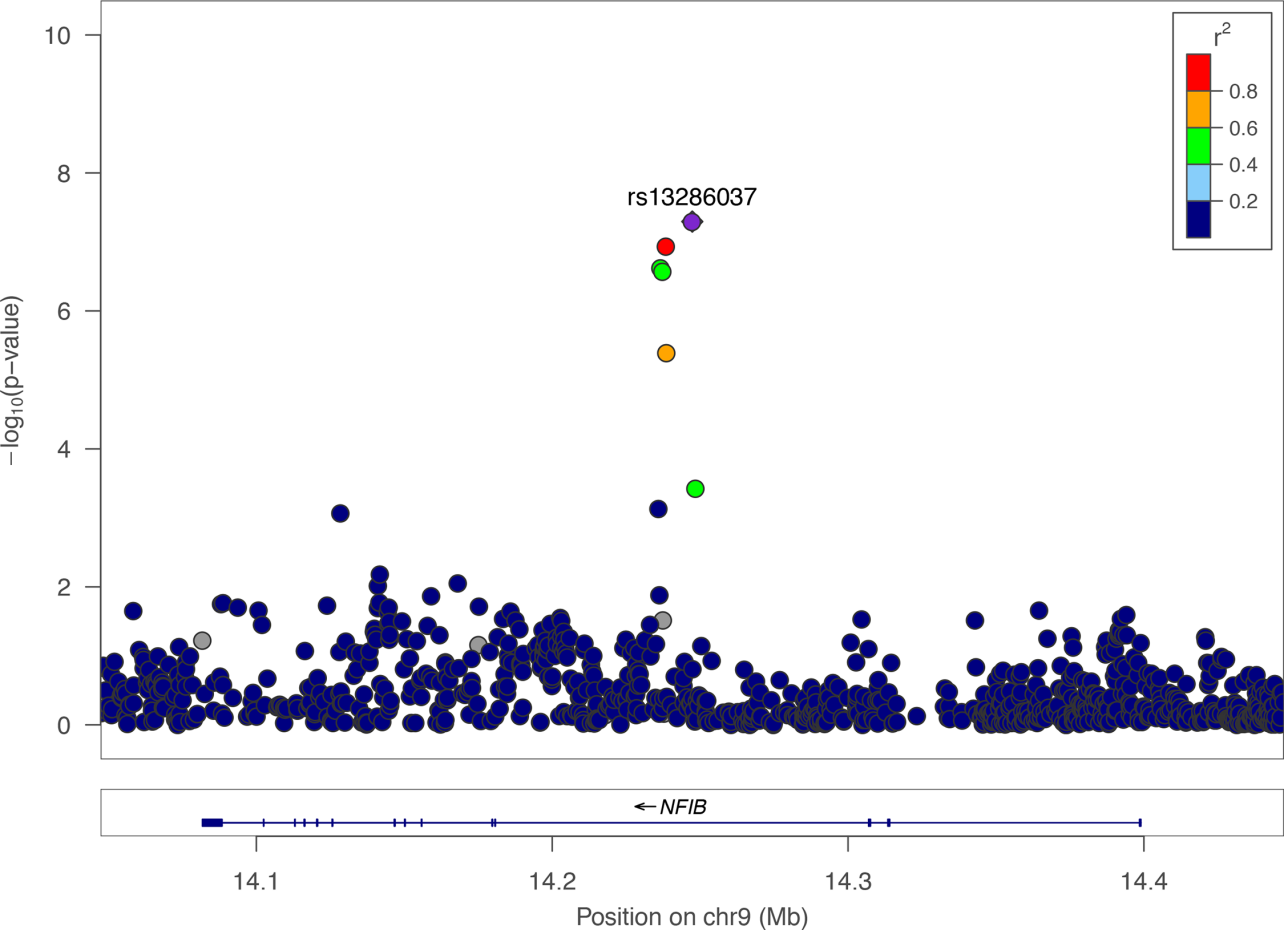
Regional-association plot for rs13286037 with ankle injury. Tested SNPs are arranged by genomic position on chromosome 9 (x-axis) in a 400 kb window around the lead SNP rs13286037 (purple diamond). The y-axis indicates -log_10_ p-values for association with ankle injury for each SNP. rs13286037 is located in the intron of *NFIB*. The color of dots of the flanking SNPs indicates their linkage disequilibrium (R^2^) with the lead SNP as indicated by the heat map color key. Red dot indicates rs35128680, which is tightly linked to rs13286037 (R^2^ = 0.99).

chr21:47156779:D is located in the intergenic region between the protein-coding genes *COL18A1*, *SLC19A1* and *PCBP3* on chromosome 21 (Fig 3). *COL18A1* encodes the alpha chain of type XVIII collagen, which is a structural component of tendons and ligaments [16]. *SLC19A1* encodes Solute Carrier Family 19, which transports folate into cells [17]. *PCBP3* encodes Poly(rC)-Binding Protein 3, which binds poly(C) stretches in RNA [18]. rs13286037 is located in an intron of *NFIB*, which encodes a transcriptional repressor protein (Fig 4)[19].

We searched for a mechanism whereby these SNPs or a linked SNP might affect the activity of nearby genes to account for their effects on ankle injury. Using R^2^>0.7 as a threshold, there are three other SNPs (rs76694187, rs138382277 and rs118069956) that are in the same linkage disequilibrium block as chr21:47156779:D spanning about 71 kb on chromosome 21 (S1 Table). Any one of these four SNPs might be responsible for affecting risk for ankle injury, with the others showing an association simply due to linkage.

None of the four SNPs in the linkage block on chromosome 21 (chr21:47156779:D, rs76694187, rs138382277 and rs118069956) are in a coding region (Fig 3). ChIP seq experiments from ENCODE indicate that rs118069956 is located within a binding site for the transcription factors GATA2 and REST, raising the possibility that variation at rs118069956 might alter binding of these transcription factors and thereby influence expression of nearby genes [20]. rs118069956 is also located in a DNAse I hypersensitive region, which is a region of open chromatin often caused by binding of transcription factors [20]. However, gene expression experiments have not yet been able to show that allelic variation in rs118069956 leads to changes in expression of nearby genes [21]. A second SNP in this region (rs138382277) might be responsible for variation in expression of a nearby long non-coding RNA. The Genotype-Tissue Exchange (GTeX) Portal has shown that rs138382277 is an expression quantitative trait locus (eQTL) for *LINC00205* (Long Intergenic Non-Coding RNA); specifically, the minor allele of rs138382277 is associated with lower expression of *LINC00205* and increased risk for ankle injury. *LINC00205* is located about 350 kb from rs138382277with no known function, although this type of RNA has been found to alter chromatin structure and affect levels of gene expression [22].

For the locus on chromosome 9, rs35128680 is located 8.8 kb away from the sentinel SNP rs13286037 with R^2^ = 0.90, indicating that the genotypes at these two SNPs are highly correlated (Fig 4). Data from the ENCODE projects show that rs35128680 is located within the central portion of the binding sites for three transcription factors (SMARCC1, TRIM28, MAX) and is also in a DNAse I hypersensitive site [20]. These results suggest that rs35128680 might affect binding of one or more of these transcriptions factors, thereby changing expression of a nearby gene and affecting risk for ankle injury. However, expression data from the GTeX consortium has not yet shown that rs35128680 is an eQTL for *NFIB* or any other closely-linked gene, possibly because the relevant cell or tissue type for ankle injury has not been tested.

### Re-testing *ACTN3* R577X for association with ankle injury

Shang et al. have reported that the R577X mutation in *ACTN3* (rs1815739) showed an association with acute ankle sprains [4]. We looked up the values for this SNP in our cohort and found that it did not show any signal of association (p = 0.90; OR = 1.00; 95% CI = 0.93–1.08).

## Discussion

Ankle injuries, including sprains, strains, and other derangements, are common in athletes [2,3]. While ankle injuries are usually caused by an acute eversion or inversion, certain populations may be at greater risk of injury following such insults. Several risk factors have been described, however the genotype of the athlete may also impact an individual’s risk for ankle injury, as well as the severity of injury and rate of recovery [2,3]. Such individuals may ameliorate their risk with preventative training, tailored conditioning and appropriate footwear [23–27].

### Genetic markers for ankle injury

This study provides new information about the genetic factors associated for ankle injury. We demonstrated the first evidence for genetic factors affecting ankle injury with genome-wide significance, with large-scale genotype and phenotype data from the RPGEH with 99,342 individuals including 1,696 ankle injuries. Power calculations indicate that a cohort of this size would have about a 90% chance of detecting a SNP with an association to ankle injury at genome-wide significance (assuming genotype relative risk of 1.7, minor allele frequency of 5%).

An indel (chr21:47156779:D) showed an association with ankle injury that was genome-wide significant (3.8x10^-8^), and rs13286037 showed an association that was nearly genome-wide significant (5.1x10^-8^). A previous study found that most genetic associations with moderate p-values (defined as p≤10^−7^ and p≥5x10^-8^) were validated in subsequent studies [28]. These results should be replicated in an independent population in future studies. For chr21:47156779:D, we note that the imputed genotype was inferred with only 73% accuracy, indicating that the association with ankle injury should be viewed with some caution.

### Potential genetic mechanisms for ankle injury

The chr21:47156779:D locus on chromosome 21 contains four linked variants that are located in the intergenic region between *COL18A1*, *SLC19A1* and *PCBP3* (Fig 3). *COL18A1* stands out as it encodes a collagen protein that might have a structural role in tendons or ligaments in the ankle [16]. chr21:47156779:D is an indel that has not been queried by either the ENCODE or GTeX project about whether it affects gene expression. rs118069956 is located 25 kb from chr21:47156779:D, and is situated in the binding region for the transcription factors GATA2 and REST [20]. A different SNP in this locus, rs138382277, is located 41 kb from chr21:47156779:D and is an eQTL for *LINC00205*, a long non- coding RNA situated 350 kb away. In summary, the association between genetic variation at this locus on chromosome 21 and ankle injury might involve changes in expression of a collagen gene or one of the two other nearby genes, or it might involve changes in expression of *LINC00205* located 350 kb away.

The locus on chromosome 9 contains two SNPs that show a moderate association with ankle injury (S1 Table). Both SNPs lie within an intron of a transcription factor gene *NFIB* (Fig 4). rs35128680 may affect expression of nearby genes as it is located within the binding regions of three transcription factors (SMARCC1, TRIM28, MAX)[20].

### Predictive power of genetic testing for ankle injury

Individuals who have one copy of the risk allele for chr21:47156779:D (A) or rs13286037 (A) have an increased risk of 1.86-fold or 1.58-fold compared to individuals lacking a risk allele in our cohort, respectively. For the general population, a 58% or 86% increased relative risk for ankle injury may not warrant a change in lifestyle. For elite athletes participating in a jumping sport, however, this level of risk may warrant attention with regard to training regimen, because the consequences of injury can be substantial.

We were not able to replicate the association of the R577X mutation in *ACTN3* (rs1815739) with ankle injuries [4]. Power calculations show that our chance for replicating this result from a cohort of 1696 cases at p≤0.05 was 95% (assuming genotype relative risk≥1.2). One explanation for the lack of validation is that the previous study looked at cases of acute ankle sprain in young, Chinese male soldiers. The difference in injury phenotype (ankle sprains/strains/derangements vs. acute ankle sprains), race (mostly European vs. Han Chinese) or population (general population in the Bay Area vs. soldiers) could account for the difference in the findings. Nevertheless, evidence from many other studies suggests that candidate gene associations need to be independently replicated, otherwise their credibility is low [29,30].

### Limitations and future directions

As noted with previous analyses of this cohort, there are several limitations to this type of study [5,11]. First, the phenotypes were defined from codes contained in the electronic health records, which may be inaccurate. 877 of the 1696 cases (52%) were identified based on four ICD-9 codes: 845.1, 845.2, 845.3 and 845.9. These four ICD-9 codes do not distinguish between ankle sprains and ankle strains. Furthermore, ICD-9 code 718.87 identified 421 cases of either ankle or foot derangements not elsewhere classified. Thus, some of the 718.87 diagnoses may have involved the foot rather than the ankle, or bony or paralytic derangements rather than sprains and strains.

Second, the ankle is composed of different ligaments, including the deltoid, lateral and tibio-fibular ligaments. It is unclear whether or not these ligaments have different aetiologies or underlying mechanisms for injury. The specific ligaments affected in each injury are usually not indicated by the ICD-9, ICD-10 or CPT codes in the electronic health record.

Third, the electronic health records do not distinguish between acute and chronic ankle injuries, which might have different aetiologies. Hence, the electronic health records do not provide knowledge about the nature or specific site of the ankle injury. The genetic association results presented here may derive from any one, or all, of the types of ankle injuries. Further investigation is warranted to study the differences in underlying genetic influences between specific ankle ligaments or chronic versus acute trauma on ankle injuries.

Fourth, the cohort included people regardless of whether or not they participated in a sport. We cannot document whether the statistical association of chr21:47156779:D and rs13286037 with ankle injury was derived predominantly from the subset of the population that were active in one or more sports.

Fifth, the number of individuals of Latin-American and East Asian ethnicity was relatively small (8,560 and 7,518, respectively). The association results for these ancestry groups are weaker than those from the European ancestry group, as would be expected due to smaller sample size. Heterogeneity analysis did not show a significant difference in the effect of either chr21:47156779:D or rs13286037 between the Latin-American or the East Asian ancestry group compared to the European ancestry group.

Sixth, the genotypes of chr21:47156779:D and rs13286037 were not directly measured but rather deduced by imputation. Care should be taken until the genetic association results can be replicated using direct genotyping of these loci in an independent cohort.

In the future, it will be important to replicate these gene association results with ankle injury in independent cohorts. Additional studies are warranted to begin to illuminate the underlying biological mechanism for the association of variation near *COL18A1*, *SLC19A1* and *PCBP3* on chromosome 21 and *NFIB* on chromosome 9 with ankle injury. It will also be interesting to perform the analysis on populations of athletes competing in sports with high rates of ankle injury, such as basketball or soccer. The results from these studies may reveal whether certain genetic polymorphisms such as chr21:47156779:D or rs13286037 could be used as diagnostic markers to help predict which athletes harbor a higher risk for ankle injury. Preventative measures could then be taken to alleviate that risk, thereby reducing the overall incidence of injury.

## Supporting information

S1 Table Linkage disequilibrium blocks on chromosomes 9 and 21. (XLS)
